# Densely packed aluminum-silver nanohelices as an ultra-thin perfect light absorber

**DOI:** 10.1038/srep39791

**Published:** 2017-01-03

**Authors:** Yi-Jun Jen, Yu-Jie Huang, Wei-Chih Liu, Yueh Weng Lin

**Affiliations:** 1 Department of Electro-Optical Engineering, National Taipei University of Technology, No. 1, Sec. 3, Chung-Hsiao E. Rd. Taipei, (106) Taiwan

## Abstract

Metals have been formed into nanostructures to absorb light with high efficiency through surface plasmon resonances. An ultra-thin plasmonic structure that exhibits strong absorption over wide ranges of wavelengths and angles of incidence is sought. In this work, a nearly perfect plasmonic nanostructure is fabricated using glancing angle deposition. The difference between the morphologies of obliquely deposited aluminum and silver nanohelices is exploited to form a novel three-dimensional structure, which is an aluminum-silver nanohelix array on a pattern-free substrate. With a thickness of only 470 nm, densely distributed nanohelices support rod-to-rod localized surface plasmons for broadband and polarization-independent light extinction. The extinctance remains high over wavelengths from 400 nm to 2000 nm and angles of incidence from 0° to 70°.

The complete absorption of light is highly desirable for many applications, including the conversion of solar energy to fuel and electricity^1^,^2^. Strong light absorption requires not only high extinction but also low reflectivity to ensure the coupling of most of the incident light into the adsorbing material and the consequent dissipation of electromagnetic wave energy. A perfect light absorber is an opaque structure that exhibits low reflectivity over wide ranges of wavelength and angle of incidence. In pursuit of a compact structure to absorb light with high efficiency, metals have been used form new structures that absorb light through localized and delocalized surface plasmon resonances. Although absorption bands in such structures as metallic gratings^3^,^4^, nanoparticles^5^,^6^ and sub-wavelength slits^7^,^8^ have been improved by the use of metallic trapezoidal arrays^9^, the extinctance of such patterned layered structures cannot be maintained over 80% over all visible wavelengths. Therefore three-dimensional plasmonic structures have recently been developed to raise extinctance and extend the range of wavelengths over which high extinctance is maintained. Three-dimensional plasmonic structures with near-perfect absorption have been developed recently, but most rely on their large thicknesses to support both a graded index profile and localized surface plasmons to realize high absorption in the visible regime^10^,^11^,^12^. Recently the absorption from a three-dimensional nanohelix array has been investigated. However, those works^13^,^14^,^15^ only simulated the absorption performance of metal helix array and lacked fabrication and measurement. These structures are designed by arranging right handed and left handed metal helices alternatively on a substrate to reduce the polarization dependence. However, such alternating arrangement is difficult to be realized over a large area. For an obliquely deposited nanohelix array, the difference of absorptance between orthogonal polarized states is small. On the other hand, those simulated helices on glass substrates require at least three turns to have absorptance over 0.8. A three-turned aluminum nanohelix array with a thickness of 600 nm exhibits absorptance larger than 0.9 over a small wavelength range of around 150 nm. The absorption of a three dimensional nanohelix array at different angles of incidence is also desired to be reported. Here, glancing angle deposition^16^,^17^ is applied to fabricate a novel three-dimensional aluminum-silver nanohelix array. With a thickness of 470 nm, densely distributed nanohelices support rod-to-rod localized surface plasmons for broadband and polarization-independent light extinction. The extinctance remains high over wavelengths from 400 nm to 2000 nm and angles of incidence from 0° to 70°.

Since 2000, “perfect antireflection”, which refers to antireflection over broadranges of wavelengths and angles, has been realized practically using various nanofabrication methods^18^,^19^. A nanostructured dielectric layer with a grade-index profile that was proposed provides perfect antireflection^20^,^21^. This grade index profile has been realized using an inhomogeneous structure with a continuously varying filling factor from a dense bottom to a porous top surface to eliminate abrupt reflection from interfaces between discrete index materials. In nature, the eyes of moths exhibit such grade index profile^22^; silicon nanotip arrays have been fabricated as bio-mimic structures with perfect reflectivity^23^. An obliquely deposited five-layered structure that composed of porous SiO_2_ and TiO_2_ films also exhibits the “moth-eye” phenomenon^24^. A perfect absorber is thus defined as an opaque device that exhibits perfect antireflection. Such graded-index structures with low extinction coefficients require sufficient thicknesses to ensure low transmission^25^.

For the past ten years, relevant research has focused on the development of highly efficient light absorbers, in which a minimal amount of material that absorbs all incident light. Noble metals with strong, broadband and weakly polarization-dependent light extinction are promising candidates for use as components to enhance the absorption of electromagnetic waves. Some efforts have been made to develop sandwiched structures with metal particle arrays^26^,^27^ or patterned metal surfaces^28^,^29^ as the top layer to excite plasmons among the three-layered structures, suppressing reflectivity. However, broadband and wide-angle absorption have not yet been achieved simultaneously for these sandwiched structures.

One-dimensional plasmonic structures have recently been fabricated by alternatively arranging metal and dielectric films as a metal-dielectric multilayer. The thickness and refractive index of each layer are designed to yield a stratiform structure with an equivalent admittance of approximately unity in a designated range of wavelengths^30^,^31^. However, the absorption is limited by the bandwidth of admittance matching. Two-dimensional gold convex grooves^10^ and a three-dimensional metal- coated or metal nano-tip array^11^ yield a filling fraction of metal that is proportional to the depth from the top surface. The low density of the metal around the top layer has an effective refractive index that is close to unity to minimize reflectivity. The effective extinction increases with the proportion of metal and depth. Since the effective medium approximation is valid only for a low filling fraction of metal, when the fraction of metal exceeds some critical value, localized surface plasmon resonance occurs around the narrow gaps between metal clusters where photons are coupled to conduction electrons in the metal and the local field is enhanced. Such a plasmonic ‘moth-eye’ structure requires a thickness of approximately 9 μm to achieve absorptance of over 90% in a range of wavelengths from 300 nm to 1200 nm^11^.

In this work, the glancing angle deposition method is utilized to sculpt a three-layered nanohelix array. A metal nanohelix array can reportedly be obliquely deposited on a seeded substrate by spinning the cooled substrate during deposition^32^. Our recent work has shown that silver and gold nanohelix arrays can be obliquely deposited on a smooth substrate at with an optimal spin rate with respect to the deposition rate^33^,^34^. Without patterned substrates, nanohelices are grown by self-shadowing; the nanohelices are distributed densely, and rod-to-rod gaps with widths of less than 10 nm are randomly and densely distributed within the array. The properties of a nanohelix, including pitch length, radius of curvature, arm width, and pitch angle depend not only on the deposition conditions but also on the species of the evaporated metal. For a particular deposition angle and deposition rate, silver and aluminum nanohelixes grow with different morphologies and their spiral structures are optimized at different substrate spin rates. Aluminum nanohelices have a longer pitch and a smaller radius of curvature than silver nanohelixes. Therefore, silver naonhelixes are capped with aluminum nanohelixes to form an Al-Ag nanohelix array. Such a plasmonic array absorbs light efficiently over wide ranges of frequencies and angles of incidence.

Nanohelices were deposited in an electron evaporation system; during this process, the substrate normal was tilted at an angle of 86° from the direction of incidence of the vapor. The center of the substrate and the evaporation source were vertically separated by 290 mm. Liquid nitrogen was passed through a loop under the substrate to cool the substrate holder to −140 °C. A background pressure of 4×10^−6^ torr was achieved by pumping before evaporation. The deposition rate was maintained at 0.3 nm/s. The rate of rotation of the substrate was varied to match the deposition rate and thereby optimize the helical nanostructure. The optimum spiral silver and aluminum nanohelix arrays were deposited at substrate spin rates of 0.017 rpm and 0.035 rpm, respectively. Figure 1(a,b) shows the morphologies of both one-turn silver and aluminum nanohelix arrays. Since the low melting point of aluminum results in high adatom mobilities that allow very high diffusion rates, the structure of Al nanohelices did not develop as well as that of Ag nanohelices. Table 1 compares the pitch lengths (P), radii of curvature (R), armwidths (W) and pitch angles (φ) of both arrays. The mean pitch angle, 50°, and pitch length, 265 nm, of Al the nanohelix array were larger than those of the Ag nanohelix array. The mean radius of curvature, 90 nm, and arm width, 60 nm, of the Al nanohelix array were smaller than those of the Ag nanohelix array. The Al nanohelixes were then deposited upon the Ag nanohelixes under the aforementioned deposition conditions to form a two-turn nanohelix array, as shown in Fig. 1(c). Since the bottom Ag nanohelices functioned as a seed layer, the Al nanohelices with mean (*P, R, W, φ*) = (253 nm, 80 nm, 70 nm, 45°) effectively developed. The low pitch angle of Ag nanohelices caused large radius of curvature. With increasing thickness, the Ag rods became broad and inclined to connect to adjacent rods. In order to keep most helices close but separately, one-turn Al nanohelices with high pitch angle then grew upon the one-turn Ag helices. Therefore the Al nanohelices with small radius of curvature on the Ag nanohelices support gaps between helices from the bottom to the top of the Al-Ag nanohelix array.

The reflectance (*R*) and transmittance (*T*) of the Al-Ag nanohelix array aremeasured using a Hitachi 4100 spectrophotometer. Extinctance (*E*) is defined as 1 − *R* − *T*. Figure 2 schematically depicts an Al-Ag nanohelix whose z axis is normal to the surface and whose y-z plane is coincident with the initial deposition plane that contains the surface normal and the direction of initial deposition flux. Figure 3 shows the experiment and simulation of x-polarized and y-polarized spectra (*T, R*, and *E*) obtained at normal incidence. Both *T* and *R* depend only weakly on polarization and the difference between the values of *T*(*R*) in orthogonal linear states is less than 10%. The average extinctance exceeds 93% of the incident light over a wide range of wavelengths from 400 nm to 2000 nm. The measured transmittance is less than 1% in the visible range, rising to around 9% in the near infrared range. The measured reflectance remained around 5% in the visible range, dropping to around 3% in the near infrared range. The nanohelices that we adopted in simulation were sampled from the SEM images. Since the helices are irregularly distributed on the substrate, a certain discrepancy appears between simulation and measurement. Although the discrepancy between simulated and measured extinctance spectra is around 10% at wavelengths over 1500 nm in Fig. 3(c), the average discrepancy over the whole wavelengths is only 4.4%. Figure 4 shows p-polarized and s-polarized extinctance measured at oblique incidence on the plane of incidence (x-z plane). The wavelength ranges from 400 nm to 2000 nm and the angle of incidence ranges from 20° to 70°. The optical properties include wide-angle and broadband antireflection as well as strong light absorption. Both p- and s- polarized extinctance values exceed 90% at angles of incidence of less than 45°. From the p-polarized spectrum, strong light extinctance of over 90% is maintained over wavelengths from 400 nm to 2000 nm and angles of incidence from 20° to 65°. Notably, extremely strong light extinctance of over 97% is maintained at wavelengths from 800 nm to 1100 nm and angles of incidence from 20° to 55°. From the s-polarized spectrum, a strong light extinctance of over 90% is maintained at wavelengths from 400 nm to 2000 nm and angles of incidence from 20° to 45°. The average extinctance of both polarization states over the entire range of measured wavelengths for light of either polarization decays from 92% at an angle of incidence of 55° to 77% at an angle of incidence of 70°.

Strong light absorption arises from the strong light coupling by top Al nanohelixes and the dissipation of light by Al and Ag nanohelixes. Some recent investigations have demonstrated that an upright aluminum nanorod array^35^,^36^ exhibits weakly polarization-dependent light coupling in the visible regime over a wide range of angles of incidence. In a high-density aluminum nanorod array^36^, when rod-to-rod distance is comparable or less than the nanorod size, near-field interactions are expected to be significant and bandwidth of extinction is extended. The enhanced light trapping and surface plasmonic property that originates from the nonperiodic, non-uniformly sized, and closely spaced structure of the nanorods promotes rod-to-rod plasmonic coupling, and causes low reflectance and high optical absorption. The top Al nanohelix array exhibits low reflectivity and dissipates light energy. Competition during shadowing deposition yields incomplete nanohelixes which were shielded from the vapor from the evaporation source during growth process. Therefore, incomplete helixes are distributed within the Al and Ag nanohelix arrays. The nano-scale gaps between the nanohelices distributed from the top to the bottom. The actual morphology of the nanohelixes is obtained from the SEM images of Al-Ag nanohelixes and used in near-field simulation. Full-field electromagnetic simulations of the Al-Ag nanohelices are conducted, and the results obtained at normal incidence are plotted in Fig. 5. The finite-difference time-domain (FDTD) (Lumerical FDTD Solutions 8.11.337) simulations involved metallic shapes that were obtained from digitized SEM images of a 400 nm × 400 nm area of experimentally fabricated sample. An unpolarized light wave with broadband wavelengths of 400–2000 nm is normally incident onto the Al-Ag nanohelices and the electric field intensity defined as |*E*/*E*_*i*_|^2^ where *E*_*i*_ and *E* are the amplitudes of incident electric filed and localized electric field, respectively. Remarkably good agreement was achieved between the experiments and simulations.

To gain insight into the localized surface plasmon resonance and the broadening of the extinction spectra by the nanostructure of the Al-Ag helix array, the electric field profiles upon excitation by unpolarized light were calculated. The simulation involved an incident wave whose electric field had amplitude of unity and a wavelength of 550 nm. Figure 6 plots electric field intensity distributions on a logarithmic scale in cross-sections (x-y plane) at intervals of 50 nm above the substrate. The electric field is strongly localized between narrow gaps between helices in the dielectric region between the top and bottom metallic layers. The electric field intensities are calculated at four wavelengths (400 nm, 435 nm, 650 nm, and 1000 nm) and presented in the supplementary information. The field intensity distributions in the cross-sections reveal the mechanism of light absorption. The enhancement of the field on the top surface is weak so the light is coupled into the array with low reflectivity. At depths of z = 300 nm and z = 350 nm, field enhancement is strong within the narrow gaps among Al rods. Although the field decays with depth, the hot spots within the gaps among Ag rods have similar intensity to those in the top Al layer. At a wavelength of 1500 nm, the field intensity distributions shown in Fig. 7 indicate that the hot spots uniformly distributed over the whole structure. The hot spots with field intensity larger than at a wavelength of 1500 nm, the field intensity distributions shown in Fig. 7 indicate that the hot spots uniformly distributed over the whole structure. The hot spots with field intensity larger than the normalized value 32 exist on each cross-section.

In conclusion, an Al-Ag nanostructure array was constructed using glancing angle deposition. The three-dimensional plasmonic nanostructure with a thickness of only 470 nm exhibits broadband and wide-angle light extinctance. Nano-scale gaps densely distribute within these metal helices support multiple resonance for different wavelengths, polarization states and angles of incidence. Based on this work, localized surface plasmon resonances can be designed in a compact nanostructure for applications including surface enhanced Raman scattering sensing, thermal emitters, and high efficient solar cells. The fabrication method we proposed can be extended to involve various nanostructures that comprise different metals and dielectric materials to generate various extraordinary optical properties in the near future. The pattern-free and cooling substrate technique enables the low-cost mass production of various three-dimensional plasmonic nanostructures.

## Methods

### Electron beam evaporation

Fabrication by glancing angle deposition (GLAD) involved tilting the substrate at an angle *θ*_*v*_ with respect to the deposition flux in the electron-beam evaporation system. The chamber that contained the substrate and the targets of silver and aluminum were pumped to a base pressure of 4 × 10^−6^ Torr prior to evaporation. The vapours were directed onto a glass substrate (BK7) at a deposition angle *θ*_*v*_ = 86° with respect to the normal of the substrate plane. The deposition rate and the thickness of the Ag and Al were controlled using a quartz crystal microbalance adjacent to the substrate holder. The deposition rate was fixed at 0.3 nm/s. The evaporated source was located at 29 cm from the center of the substrate plane. During the deposition, the temperature of the substrate was reduced using liquid nitrogen cooling. The substrate mounting block was designed to carry liquid nitrogen in an open-cycle dewar configuration. Stainless steel flexible hose were used for connecting the feed-through to the substrate mounting block. The BK7 substrate was 1.5 mm thick and the substrate holder was made of cooper. A K-type thermocouple was directly mounted on the copper substrate holder for temperature measurements. The temperature of the copper sample holder was maintained approximately −140 °C.

### Optical measurements

The reflectance and transmittance of the Al-Ag nanohelix array were measured using Hitachi 4100 spectrophotometer. The spectra were measured at wavelengths from 400 nm to 850 nm with photomultiplier and wavelengths from 850 nm to 2000 nm with cooled type Pbs(Polycrystalline lead sulfide detectors). At oblique incidence, p-polarized and s-polarized spectra were measured separately at angles of incidence from 20° to 70° in steps of 5 degrees.

## Additional Information

**How to cite this article**: Jen, Y.-J. *et al*. Densely packed aluminum-silver nanohelices as an ultra-thin perfect light absorber. *Sci. Rep.* **7**, 39791; doi: 10.1038/srep39791 (2017).

**Publisher's note:** Springer Nature remains neutral with regard to jurisdictional claims in published maps and institutional affiliations.

## Supplementary Material

Supplementary Information

## Figures and Tables

**Figure 1:**
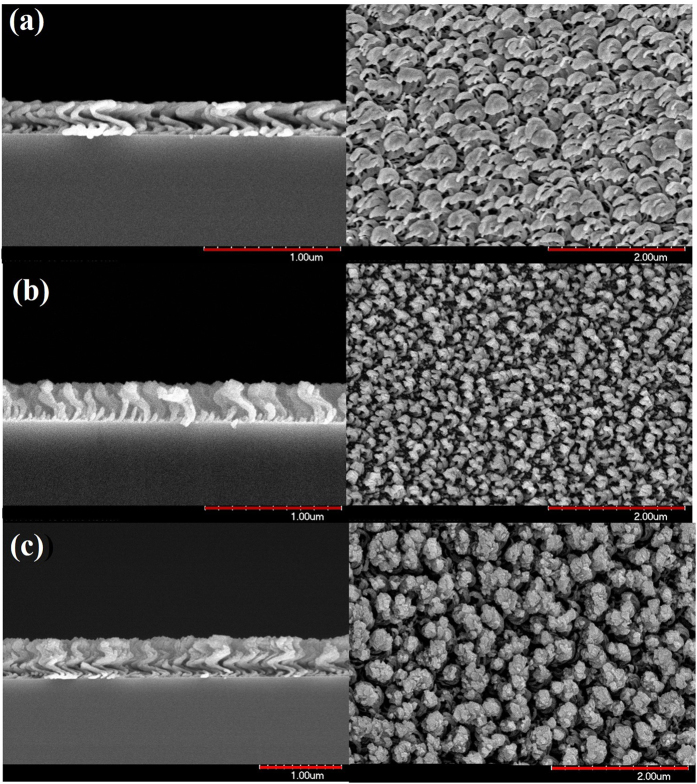
Top-view and cross-section SEM images of (**a**) Ag nanohelix array, (**b**) Al nanohelix array and (**c**) Al-Ag nanohelix array.

**Figure 2:**
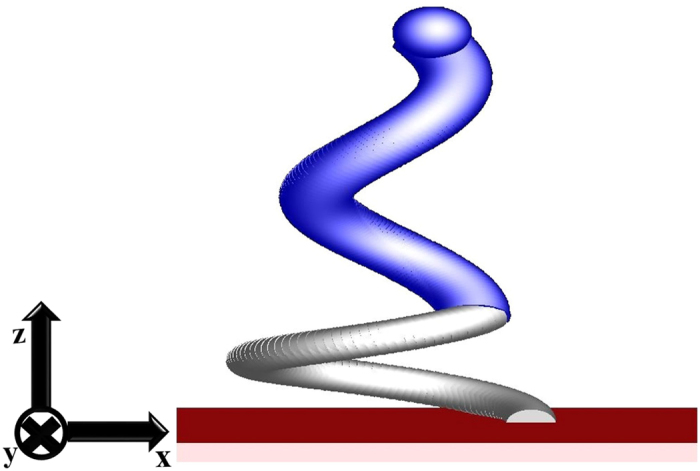
Schematic drawing of an Al-Ag nanohelix.

**Figure 3:**
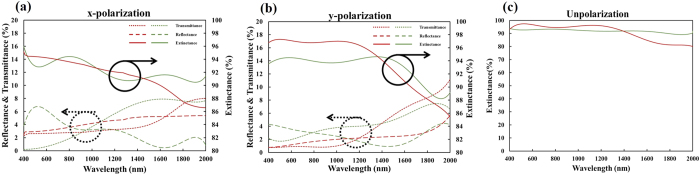
(**a**) Measured and simulated x-polarized spectra of transmittance, reflectance and extinctance. (**b**) Measured and simulated y-polarized spectra of transmittance, reflectance and extinctance. (**c**) Measured and simulated unpolarized spectra of extinctance. Measured and simulated results are shown in green and red, respectively.

**Figure 4:**
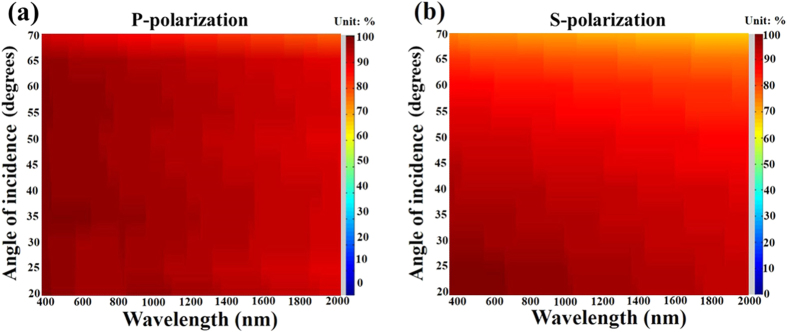
(**a**) P-polarized and (**b**) s-polarized extinctance spectra as functions of wavelength and angle of incidence.

**Figure 5:**
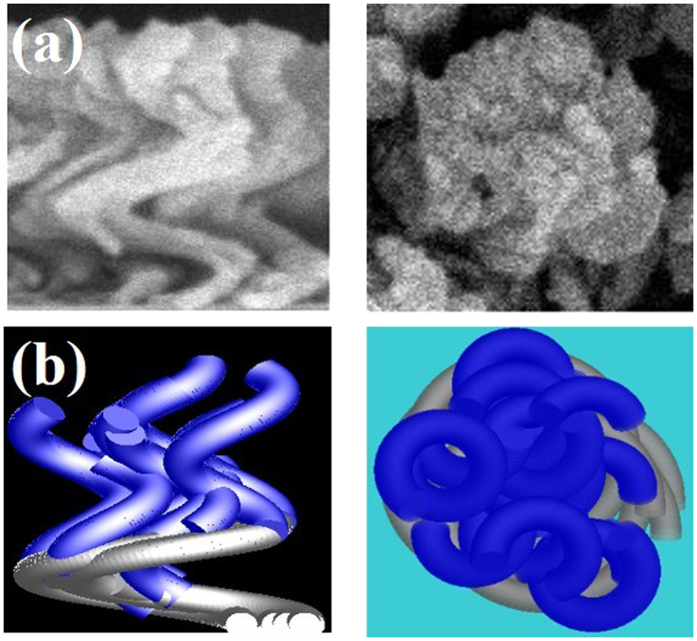
(**a**) The cross section-view and top-view SEM images; (**b**) The schematic drawing of Al-Ag nanohelixes for simulation.

**Figure 6:**
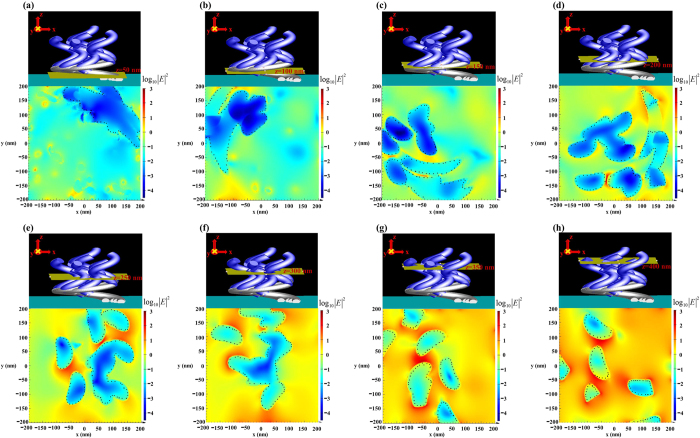
Three-dimensional view of simulated absorber structure with cross-sections at different altitudes and maps of electric field intensities on these cross-sections for the incident wavelength of 550 nm. The black dot lines indicate the boundary between metal and air.

**Figure 7:**
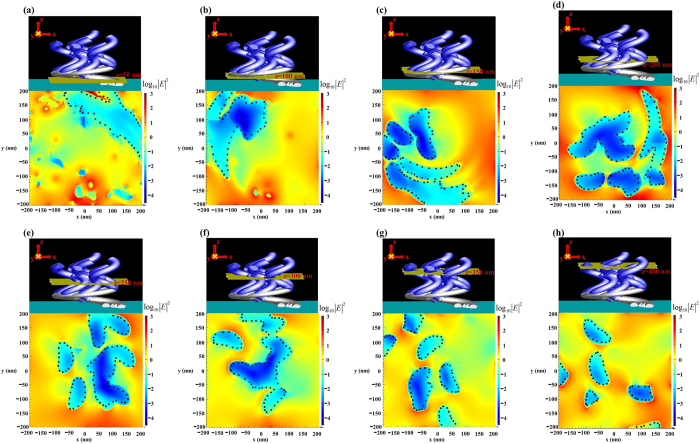
Three-dimensional view of simulated absorber structure with cross-sections at different altitudes and maps of electric field intensities on these cross-sections for the incident wavelength of 1500 nm. The black dot lines indicate the boundary between helix and air.

**Table 1:** Spin rate, radius of curvature, arm width, pitch length, and pitch angle of a metal nanohelix array.

	Spin rate (ω)	Radius of curvature (*R*)	Arm width (*W*)	Pitch length (*P*)	angle (*φ*)
Al	0.035 rpm	90 nm	60 nm	265 nm	50°
Ag	0.017 rpm	160 nm	50 nm	183 nm	25°
