# Supplementary material for: Densely packed aluminum-silver nanohelices as an ultra-thin perfect light absorber
Source: Sci Rep. 2017 Jan 3;7:39791. doi: 10.1038/srep39791 (PMC5206654; doi:10.1038/srep39791)
Supplement: Supplementary Information [file srep39791-s1.pdf]

**Supplementary Information:**

# Densely packed aluminum-silver nanohelices as an ultra-thin perfect light absorber

Yi-Jun Jen[1]*, Yu-Jie Huang[1], Wei-Chih Liu[1], Yueh Weng Lin[1]

[1] Department of Electro-Optical Engineering, National Taipei University of Technology, 10608 No. 1, Sec. 3, Chung-Hsiao E. Rd. Taipei, Taiwan

*Corresponding Email: jyjun@ntut.edu.tw

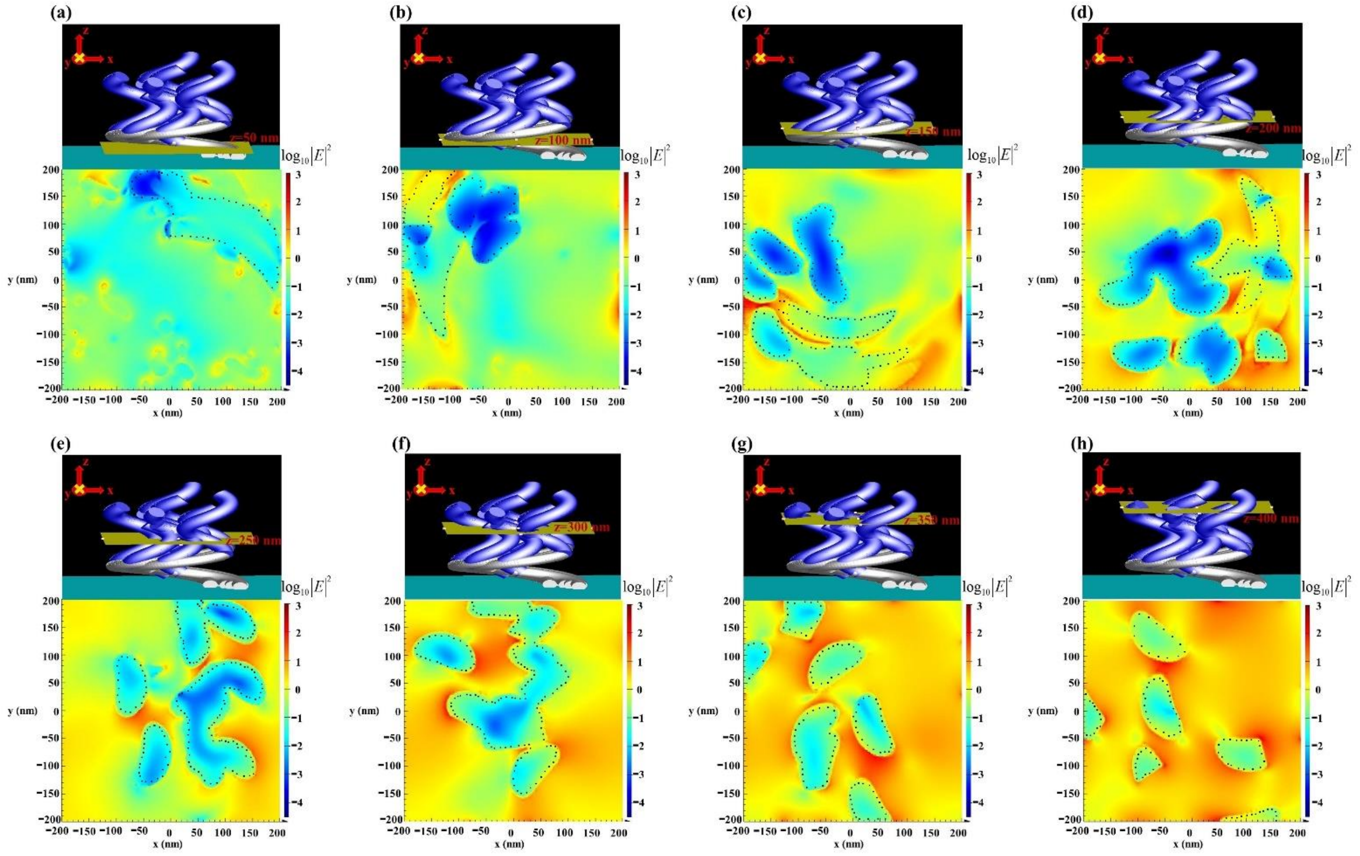


**Figure S1.** Three-dimensional view of simulated absorber structure with cross-sections at different altitudes and maps of electric field intensities on these cross-sections for the incident wavelength of 400 nm. The black dot lines indicate the boundary between metal and air.

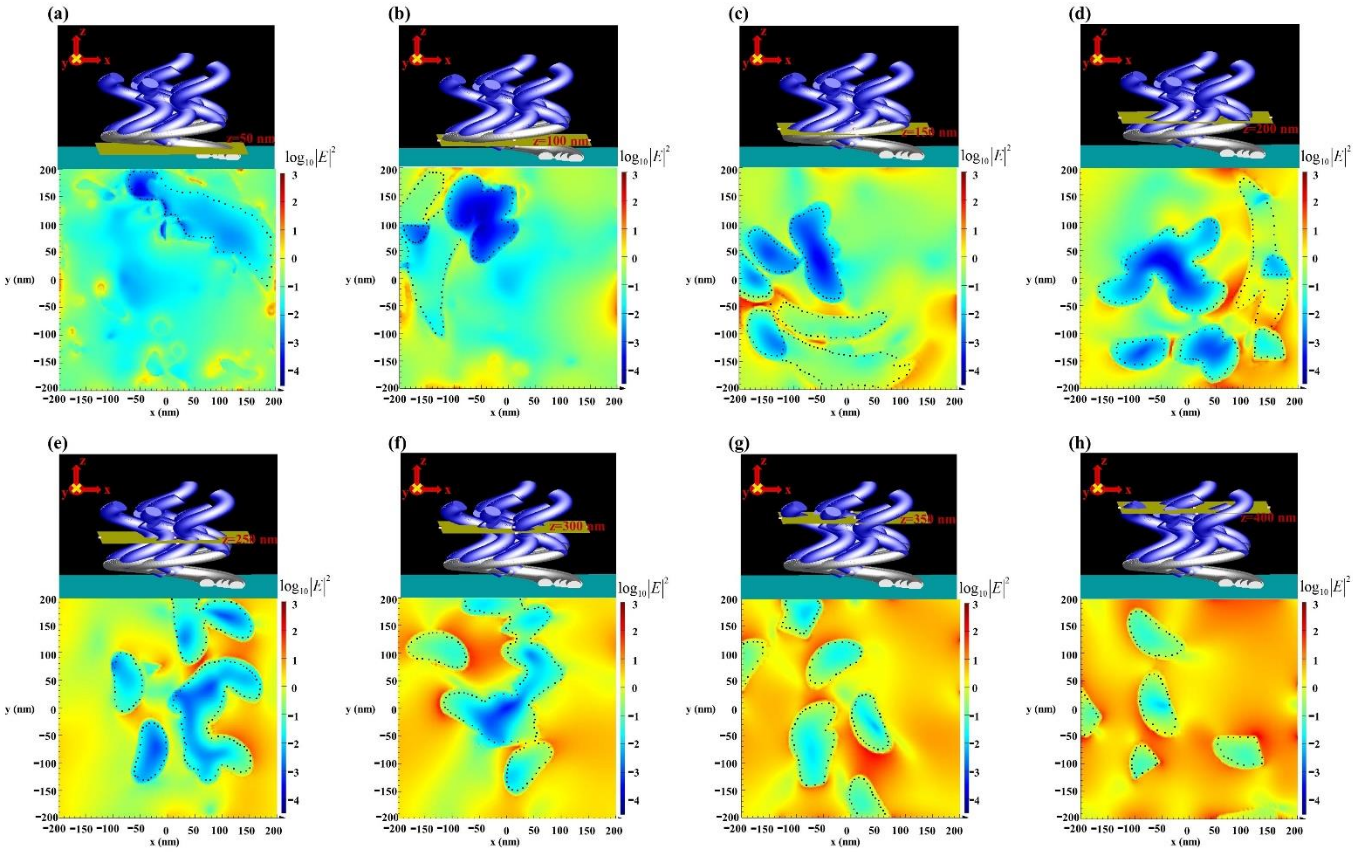


**Figure S2.** Three-dimensional view of simulated absorber structure with cross-sections at different altitudes and maps of electric field intensities on these cross-sections for the incident wavelength of 435 nm. The black dot lines indicate the boundary between metal and air.

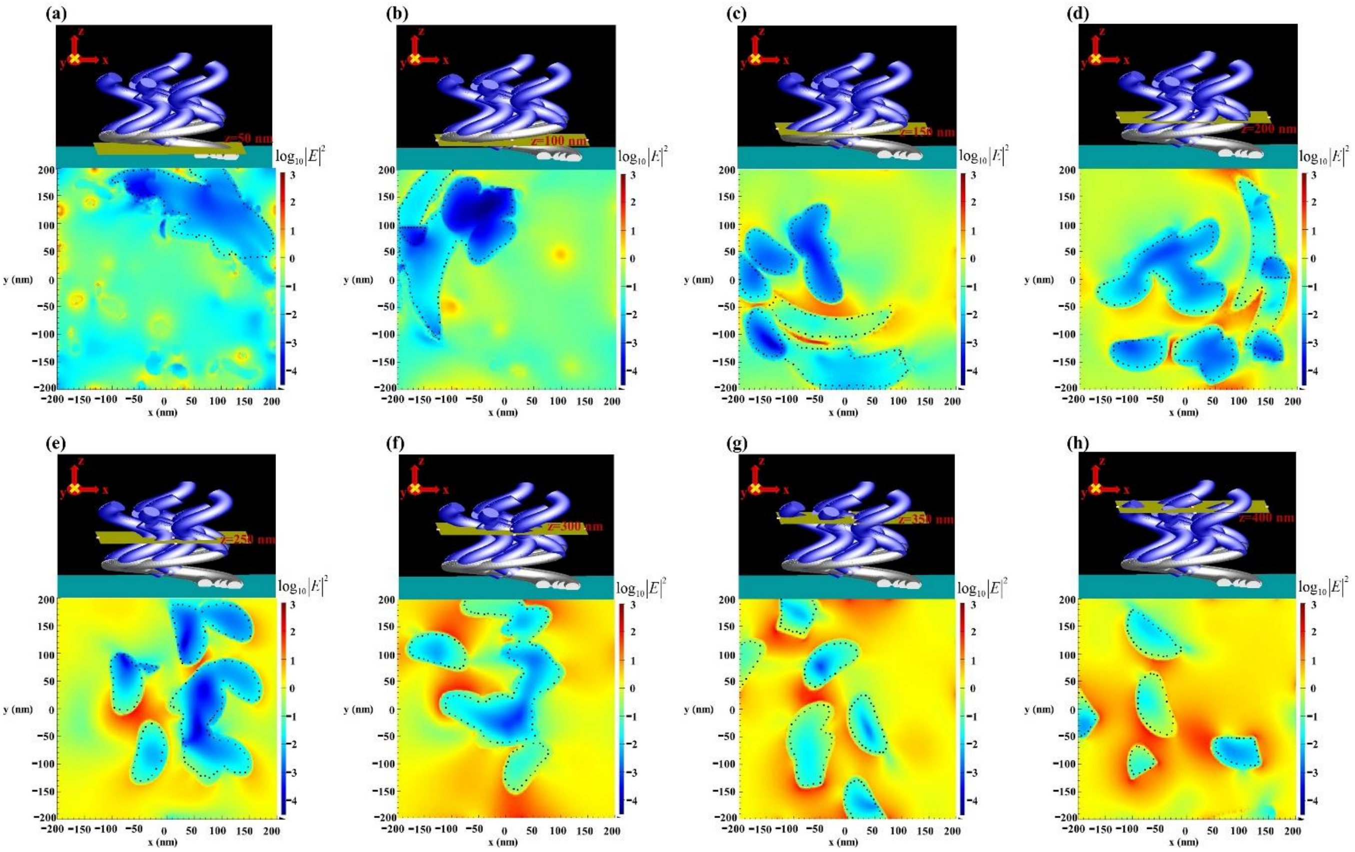

**Figure S3.** Three-dimensional view of simulated absorber structure with cross-sections at different altitudes and maps of electric field intensities on these cross-sections for the incident wavelength of 650 nm. The black dot lines indicate the boundary between metal and air.

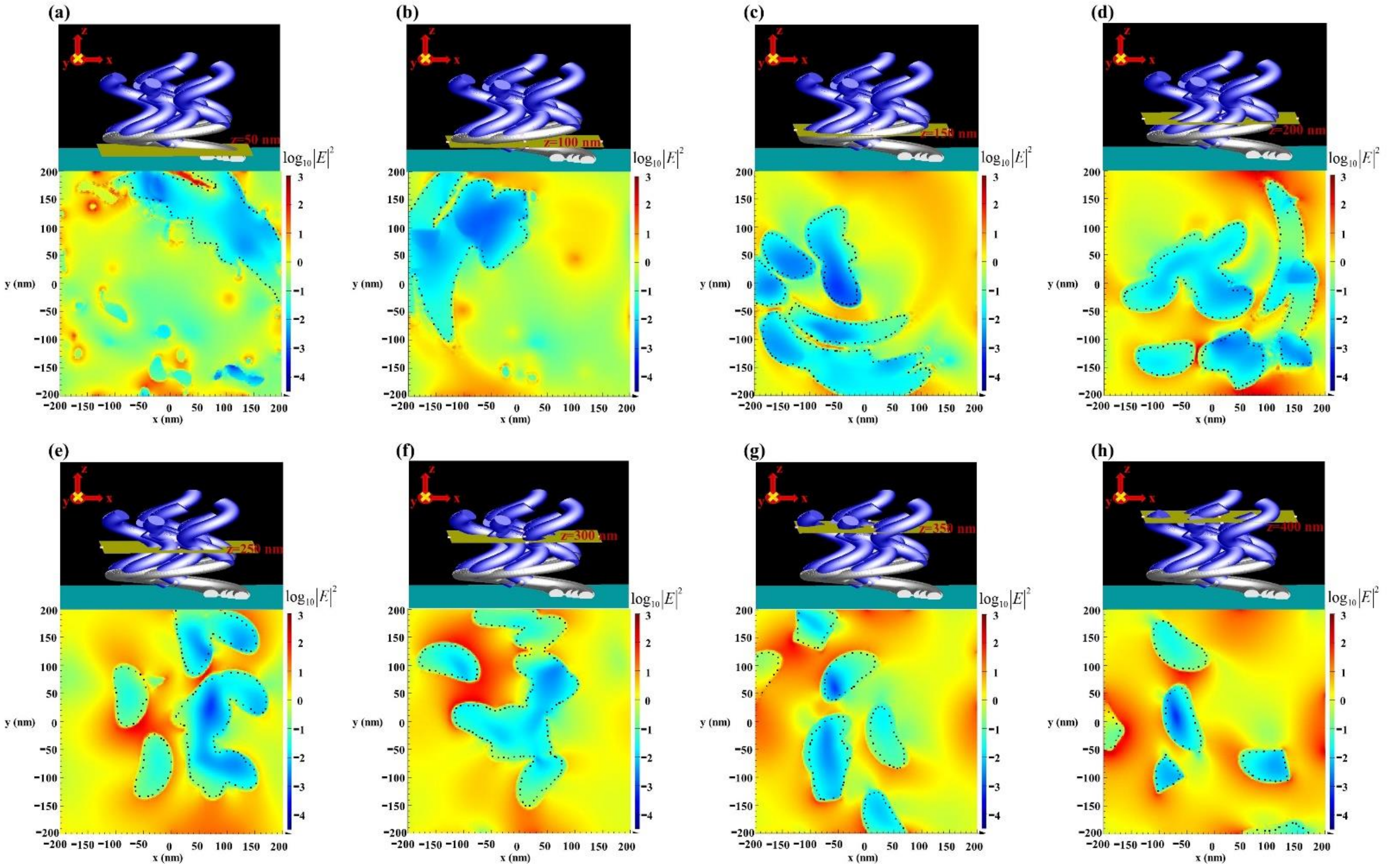


**Figure S4.** Three-dimensional view of simulated absorber structure with cross-sections at different altitudes and maps of electric field intensities on these cross-sections for the incident wavelength of 1000 nm. The black dot lines indicate the boundary between metal and air.
